# In Vitro Methodologies for Evaluating Colon-Targeted Pharmaceutical Products and Industry Perspectives for Their Applications

**DOI:** 10.3390/pharmaceutics14020291

**Published:** 2022-01-26

**Authors:** Mauricio A. García, Felipe Varum, Jozef Al-Gousous, Michael Hofmann, Susanne Page, Peter Langguth

**Affiliations:** 1 Department of Biopharmaceutics and Pharmaceutical Technology, Johannes Gutenberg University Mainz, 55099 Mainz, Germany; magarcia@uni-mainz.de (M.A.G.); joalgous@uni-mainz.de (J.A.-G.); 2 Pharmaceutical Research and Development, F. Hoffmann-La Roche Ltd., 4070 Basel, Switzerland; felipe.varum@roche.com (F.V.); michael.hofmann.mh5@roche.com (M.H.); susanne.page@roche.com (S.P.); 3 Department of Pharmaceutical Sciences, University of Michigan, 428 Church Street, Ann Arbor, MI 48109, USA

**Keywords:** colon targeting, modified drug release, pH-dependent release, enzymatic triggered release, bio-relevant dissolution, quality control, bio-predictive dissolution

## Abstract

Several locally acting colon-targeted products to treat colonic diseases have been recently developed and marketed, taking advantage of gastrointestinal physiology to target delivery. Main mechanisms involve pH-dependent, time-controlled and/or enzymatic-triggered release. With site of action located before systemic circulation and troublesome colonic sampling, there is room for the introduction of meaningful in vitro methods for development, quality control (QC) and regulatory applications of these formulations. A one-size-fits-all method seems unrealistic, as the selection of experimental conditions should resemble the physiological features exploited to trigger the release. This article reviews the state of the art for bio-predictive dissolution testing of colon-targeted products. Compendial methods overlook physiological aspects, such as buffer molarity and fluid composition. These are critical for pH-dependent products and time-controlled systems containing ionizable drugs. Moreover, meaningful methods for enzymatic-triggered products including either bacteria or enzymes are completely ignored by pharmacopeias. Bio-predictive testing may accelerate the development of successful products, although this may require complex methodologies. However, for high-throughput routine testing (e.g., QC), simplified methods can be used where balance is struck between simplicity, robustness and transferability on one side and bio-predictivity on the other. Ultimately, bio-predictive methods can occupy a special niche in terms of supplementing plasma concentration data for regulatory approval.

## 1. Introduction

Colon-targeted formulations have been developed and commercialized over the past decades mainly aiming at local therapeutic action for the treatment of colonic diseases (e.g., inflammatory bowel disease or colon cancer). More recently, colonic targeting is gaining more attention with ambitious goals of achieving local as well as systemic delivery of some compounds, which could be degraded in the stomach and the small intestine, such as therapeutic proteins and other novel format molecules (e.g., oligonucleotides). Numerous approaches have been attempted for this purpose each with its advantages and disadvantages from a biopharmaceutic point of view.

Locally acting drug products present a particular challenge when it comes to biopharmaceutic assessment. Bioavailability is defined as “the rate and extent to which the active ingredient or active moiety is absorbed from a drug product and becomes available at the site of action” [1]. The traditional method for bioavailability assessment is based on using plasma drug concentration as a surrogate indicator for drug availability at the site of action. This is justified by the fact that drug molecules need to reach systemic circulation before arriving at the site(s) of action.

However, drug products with local colonic therapeutic action do not fit here [2]. This is because a drug molecule will reach its site(s) of action before having entered into systemic circulation. Accordingly, venous plasma concentrations present a rather suboptimal indicator of bioavailability in this case. In addition, drug colonic luminal concentration measurements are rather complex and can yield a high variability. Thus, suitability of in vitro testing methods to predict in vivo behavior of modified release dosage forms cannot be overstated.

The complexity of the colonic environment on one hand, and the diversity of the physico-chemical principles of different formulation approaches on the other, make the design of a general one size fits all in vitro testing method unfeasible in this case. Diverse methods have been described for different colon-targeted products with varying degrees of success. The aim of this review is to discuss the current state of knowledge in this regard, while also using their strengths and limitations as starting points to explore prospects for further improvement.

## 2. Gastrointestinal Physiology: “Bypassing the Upper Gastrointestinal Tract and Targeting Distal Release”

### 2.1. Stomach and Upper Small Intestine

The human physiology of the upper gastrointestinal (GI) tract favors the absorption of food and nutrients, while preventing systemic exposure to bacteria and/or toxins. The stomach provides an inhospitable acidic environment for pathogens, which also promotes the degradation of potential toxins, in the form of peptides or proteins, by the action of digestive enzymes. In spite of the evident survival benefits of these conditions, the stomach represents a first barrier to the oral administration of new promising locally acting colonic therapeutic agents, especially labile macromolecules. Stomach pH after water ingestion ranges from 1.6 to 2.7 [3,4,5], but it may reach even higher values in some subjects [6]. These values are consistent with mass balance calculations on [H^+^] considering the concentrations of chloride, sodium, potassium and calcium were reported to be 102, 68, 13.4 and 0.6 mM, respectively (ionic strength = 100 mM) [7]. Protein content was determined at 1.8 mg/mL [7], mainly consisting of digestive proteolytic enzymes such as pepsin [8]. The resting water volume of the stomach was determined to be between 25 [9] and 45 mL [10,11], which increased abruptly after the intake of a glass of water up to 250 mL [9,10]. The water is afterwards rapidly emptied following apparent first-order kinetics (exponential decline with a constant half-life (t_1/2_) ranging from 11 to 15 min, such that more than 85% of the fluid leaves the stomach in around 35 min [3,10]. This water emptying may contribute to the emptying of microparticulate systems (<1 mm). By contrast, monolithic (non-disintegrating) dosage forms need assistance from high-pressure propagating waves to reach the small intestine, which are mediated by the inter-digestive migrating motor complex (IMMC). In a fasted stomach, the strongest waves take place during the phase III of the IMMC, which lasts less than 20 min, and are cyclically repeated every 2 h. Therefore, this latter time might represent the theoretical longest gastric residence for enteric coated colon-targeted drug products in fasted subjects [12]. However, this time may be even longer under fed conditions due to the interruption of the IMMC pattern in order to allow the disintegration of the meal, which slows the gastric-emptying rate [12].

Once emptied, luminal contents reach the upper small intestine where both, longer transit times (mean 3.5 ± 1.0 h) [13] and enhanced surface area (32–140 m^2^) [14,15], promote the absorption process. Unlike gastric residence, intestinal transit remains mostly invariable regardless of the ingested material, e.g., food, tablets, pellets or fluids [16,17]. However, small intestine transit time may be prolonged, particularly in inflammatory bowel disease patients [18]. Moreover, the intestinal transit of non-digestible materials is not a continuous process, as it alternates between static and dynamic periods [19]. These features represent challenges for colonic targeting dosage forms, particularly for those relying on transit time, because an early release may result in absorption into systemic circulation, which eventually reduces the efficacy and increases the risk of adverse effects (dose dumping). Luminal water is discretely distributed in small pockets and it occupies only a small fraction of the intestinal volume (total water volume = 43–105 mL) [10,11]. Median duodenal pH is around 6.1 [5], consistent with other reports on the proximal small intestine pH (6.0–6.2) [4,20]. The pH rises up to 6.8 in the mid small intestine [20], due to neutralizing action of bicarbonate secreted by epithelial cells [21]. In healthy subjects, bicarbonate concentrations in duodenum and jejunum are 6.7 and 8.2 mM, respectively [22,23]. Nevertheless, they may vary in jejunum between 2–10 and 6–20 mM, as well [24]. Luminal bicarbonate contributes to small intestinal buffer capacities between 3.2 and 5.6 mmol/L/ΔpH [8,25], although values as high as 13 mmol/L/ΔpH have also been reported [26]. However, continuous bicarbonate secretion, together with the relatively rapid permeation of CO_2_ (neutralization product, see Section 4.2.1) through the intestinal epithelium, enhance the luminal buffer capacity [27]. Furthermore, jejunal concentrations of sodium, chloride, potassium, and calcium are 142, 126, 5.4 and 0.5 mM (ionic strength = 139 mM), respectively [7]. The gall bladder secretes bile salts into the duodenum, resulting in total luminal concentrations of 2.6–2.9 mM [7,8], mainly corresponding to taurocholate, glycolate and glycochenodeoxycholate [26]. Additionally, human secreted enzymes in proximal small intestine (2.1–3.1 mg/mL) [7,8], namely amylase (100–150 U/mL), lipase (100–400 U/mL) and trypsin (20–50 U/mL) are secreted into the duodenum and mediate the digestion of carbohydrates, lipids and proteins, respectively [16].

### 2.2. Ileum, Caecum and Colon

Small intestinal transit ends upon the distal ileum, where undigested material is accumulated before being transferred into the large bowel. Typically, segmental transference from the small into the large intestine occurs due to propagating pressure waves which, in turn, stimulates defecation (ileocecal reflex) [28]. Pressure-sensitive non-digestible telemetric capsules sensed peak pressures of 60 mbar at the ileocecal junction [29]. Food consumption can trigger the ileocecal reflex, causing the emptying of the small intestinal content into the colon [30]. Similarly, circadian rhythm can also influence the defecation reflex. For instance, Furukawa et al. observed an increased motility with the awakening compared to sleeping periods [31]. Consistently, subjects tended to defecate in the morning. Furthermore, it has been mentioned that other factors, such as gender, age and diseases, may affect colonic transit, as well [3,16,29].

Prior to colonic arrival, non-digested material can accumulate in the cecum for different periods of time, contributing to the overall transit time variability. Transit time along the colon is highly variable and depends on the dosage form. While monolithic tablets mean residence time was 20.3 h (95% CI = 13.4–27.2 h), multi-unit pellets resided overall longer with mean times of 32.0 h (95% CI = 19.7–44.2 h) [13]. Differences may be explained by entrapment of smaller particles within haustral folds [32]. Furthermore, non-digested colonic content can either be excreted after the next meal or stay even longer, which not only increases the colonic residence times, but also the transit time variability. This is consistent with the highly intra-individual variability reported by Weitschies et al. [19], who administered a magnetically marked non-digestible capsule to the same subject in five different experimental occasions. The movement of colonic material was discontinuous and depended on bowel motility [19]. Another function of the colon is water absorption. In fact, median volume of freely mobile water in the colon was 2 mL, ranging from 0–49 mL, as determined by magnetic resonance imaging (MRI) [33]. Similar to the small intestine, colonic water is distributed in discrete pockets. Interestingly, freely mobile water in the colon is mainly located in the ascending colon [11,33]. The median volume aspirated from the ascending colon was 22.3 mL, where 70.3% corresponded to aqueous fraction [34]. Although this latter value seems to be relatively higher than values determined from MRI studies, it may be explained by the greater fraction of water bond to the thicker colonic mucus, hence, invisible to the imaging techniques. Conversely, volumes aspirated from the ileum and caecum were both drastically smaller than ascending colon content, being 3.8 (90% liquid) and 5.0 mL (70% liquid), respectively [35].

Bicarbonate concentrations of 30 mM were determined in the ileum of healthy subjects, which is higher than in the upper small intestine [23]. This increment is in line with the shift in pH values to 7.4–7.7, as measured by telemetric capsules [4,20]. Slightly higher median pH determined in ileal and cecal aspirates (pH 8.1) may be associated with the volatilization of dissolved CO_2_ [35]. In turn, the buffer capacity in these segments was 8.9 and 19.2 mmol/L/ΔpH, respectively [35]. Upon reaching the ascending colon, the pH was measured at 6.5–6.9 [4,20] and 7.8 [34], by telemetric capsules and potentiometrically in fluid aspirates, respectively. This decrease was accompanied by a rise in the buffer capacity to 21.4 mmol/L/ΔpH [34], most likely due to bacterial production of short chain fatty acids towards more distal GI segments. Accordingly, total short chain fatty acids increased from 8.6 mM in the ileum to 32.2 and 30.9 mM in the caecum and ascending colon, with acetate being the most abundant among them (67–83%) [34,35]. Furthermore, bile salts in the ileum are reduced compared to upper small intestine, given their transporter-mediated uptake in this region. Consequently, total bile salt concentrations from ileum to ascending colon varied from 71 to 183 µM, mainly corresponding to deconjugated bile acids [35]. The modulation of bile salt levels in specific regions of the GI tract has been, for instance, explored by local colonic targeting for the treatment of irritable bowel syndrome [36]. Overall, the shift in pH and buffer concentrations along the gastrointestinal tract opens a biopharmaceutic window for colonic targeting that has been used through the application of pH-dependent coatings on oral solid dosage forms. This led to the development and commercialization of several drug products, particularly for the treatment of inflammatory bowel disease, such as Asacol^®^, Salofalk^®^, Lialda^®^ and Cortiment^®^, among others [37].

**Figure 1:**
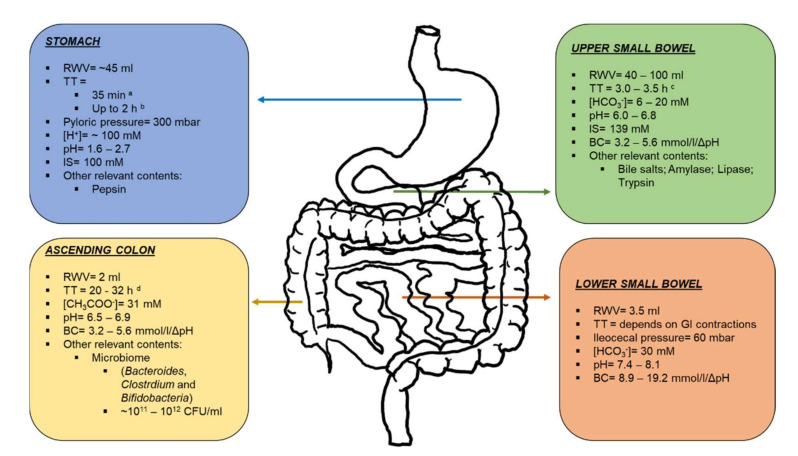
Scheme of the gastrointestinal tract describing the main physico-chemical characteristics to be considered in the development of locally acting colonic drug delivery systems. TT: transit time; RWV: resting water volume; IS: ionic strength, BC: buffer capacity. ^a^: multiparticulate dosage forms; ^b^: monolithic dosage forms; ^c^: transit time for the whole small bowel; ^d^: Mean transit times for the whole colon. References are given in text.

The gut microbiome is another feature of the GI tract that can be exploited in the development of colon-targeted products. In the stomach, the levels of bacteria are less than 10^2^ CFU/mL due in part to low gastric pH [38]. The number of bacteria increases gradually along the small intestine, but rises by several orders of magnitude beyond the ileocecal junction. This sharp rise in bacteria levels opens a biopharmaceutic window for accurate colonic drug targeting. It has been shown that the colon contains over 400 distinct species of bacteria with a population of 10^11^–10^12^ CFU/mL [38,39]. Others have postulated that the number of bacteria species may go up to 36,000, considering the inter-individual variability [40,41]. These bacteria are mainly anaerobes or facultative anaerobes; *Bacteroides*, *Clostridium* groups IV and XIV, and *Bifidobacteria* are the predominating species [42,43,44]. Colonic bacteria use undigested polysaccharides as their main source of fermentable carbohydrate [45,46] and play also a significant role in the metabolism of orally administered drugs [47,48,49]. Therefore, more flexible drug-delivery systems relying on gut microbiota have been developed, as well [50,51,52,53]. A scheme summarizing the main features of the GI tract is depicted in Figure 1.

## 3. Formulation Approaches

**Figure 2:**
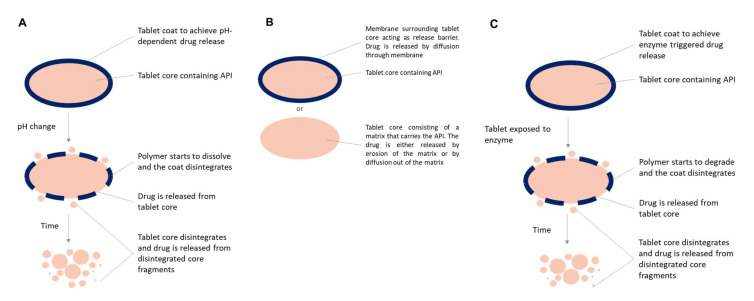
**pH-dependent systems** (**A**) using coatings from anionic polymers from methacrylic acid and methyl methycrylate (Eudragit^®^ polymers), cellulose esters hydroxypropyl methylcellulose acetate succinate (HPMC-AS), hydroxypropyl methylcellulose phthalate (HPMCP), cellulose acetate phthalate (CAP), cellulose acetate succinate (CAS), cellulose acetate trimellitate (CAT), polyvinyl acetate phthalate (PVAP), and shellac. **Time-controlled systems** (**B**) for which the release can be controlled by employing permeable insoluble polymers (top), such as ethylcellulose, or by an insoluble matrix core, such as hydroxypropyl methyl cellulose (HPMC), carnauba wax (bottom). **Enzymatic triggered release systems** (**C**) commonly use pectin, starch, alginate, gums, amylose, chitosan, dextran, chondroitin sulphate, inulin, and galactomannan embedded into a insoluble polymer (i.e., ethylcellulose) or pH-sensitive polymer (methacrylic acid and methyl methacrylate) [66,67,68,69,70].

The first approaches for targeting colonic release involved chemical synthesis of prodrugs, such as sulfasalazine, olsalazine and balsalazine. The azo-bond of the prodrug is reduced site-specifically in the colon, such that the active compound, mesalazine, is locally delivered. However, the applicability of this approach is compound-related and its scope is, therefore, limited. Nowadays, colonic targeting mostly focuses on technological approaches applied to formulations and dosage forms instead of the active compound. The different formulation strategies take advantage of GI physiology to avoid early release and effectively deliver the active pharmaceutical ingredient (API) in the colon (Figure 2). Diverse technologies to target local colonic delivery have been reviewed previously [54]. However, this review covers only the most relevant approaches in marketed technologies and drug products (Table 1), which are briefly described below.

### 3.1. pH-Dependent Systems

Most of the commercially available pharmaceutical products, such as Asacol^®^, Mezavant^®^/Lialda^®^, Salofalk^®^, Uceris^®^ targeting the colon rely on the GI pH gradient to initiate drug release (Table 1). Typically, enteric polymers such as polymethacrylates (i.e., Eudragit^®^ L100 dissolves at pH 6 or S100 dissolves at pH 7) are used in film coatings of tablets or granules to prevent premature drug release in the upper GI tract and allow release in the distal small intestine and colon (Figure 2A). Approaches relying on pH have achieved commercial success, however, there is a risk of failure, exacerbated in certain patients and conditions where the pH may be too low [55,56], or the transit time too fast [57,58], which poses a challenge for a timely and accurate colonic targeting [59,60]. In cases of too low responsiveness of traditional enteric coatings to the intestinal physiology, it can result in suboptimal performance with tablets remaining intact [61,62]. Novel technologies like Duocoat™ and OPTICORE^TM^ employ an internal buffer source from an inner coat that helps mitigating this problem [62,63]. Another technology aiming at improving the effectiveness of an enteric coating for colonic release applications is the Colopulse^®^ technology, which relies on super-disintegrants embedded into organic-based enteric coating to prompt a pulsatile release once the target pH is reached [64,65].

### 3.2. Time-Controlled Systems

Time-dependent systems can be used for sustained release products relying on the fact that the residence time in the colon constitutes the majority of the gastro-intestinal transit time (Figure 2B). Additionally, the underlying assumption is that gastric emptying and small intestinal transit has limited variability. One product example is Pentasa^®^, which is a 5-ASA product coated with an ethylcellulose-based coating (Table 1). To further improve targeting, a pH-dependent onset of the release-delaying mechanism could be added like in Salofalk^®^ and LIALDA^®^ where a pH-dependent coat surrounds a sustained release matrix core. Another formulation approach involved a time-dependent delayed release coating designed to introduce a 5–6 h onset of release delay through gradual coat erosion. The main limitation to time-dependent approaches lies in the gastrointestinal transit variability.

### 3.3. Enzymatic Triggered Release Systems

The colon harbors the largest population of gut bacteria contributing to human health and disease. This provides a great metabolic capacity that can be explored for drug-delivery approaches for colonic targeting. Of particular interest is the digestion of polysaccharides that escape digestion in the upper GI tract and can be fermented by colonic bacteria yielding lactate and short-chain fatty acids. Therefore, several formulation approaches including polysaccharides have been pursued, mostly in the form of film-coatings (Figure 2C). Many polysaccharides are widely available and are already in use as pharmaceutical excipients, which lowers the regulatory barriers in comparison to introducing a new excipient in pharmaceutical drug products. Among the most used polysaccharides are pectin and starch (and its derivatives). For instance, Sublimity Therapeutics developed a SMEDDS minisphere formulation coated with a layer of pectin and ethylcellulose which showed better colonic delivery of cyclosporine in comparison to commercially available products [71], and finally progressed into a phase 2 study, however with unsuccessful outcome. Others have investigated colonic targeting accuracy by gamma-scintigraphy in healthy subjects of pectin:HPMC compressed coating on radiolabeled tablet cores and found out that in all subjects tablets disintegrated in the ascending or transverse colon. Transit time, HPMC gelling and mechanical pressures may contribute to the different disintegration location observed [72].

Resistant starch (such as high amylose starches) and other starch derivatives (hydroxypropylated and pregelatinized high amylose starch, dextrin) have been extensively exploited for colonic targeting in the form of film coatings with insoluble polymers, such as ethylcellulose [52,53,73,74] or with enteric polymers [75,76,77,78]. These polymers prevent a premature swelling of the polysaccharide and dissolution of the coating in the small intestine. Despite promising results in vitro, in preclinical models and early clinical studies only a few systems have progressed through the pipeline. One example is the COLAL™ system comprising amylose and ethylcellulose applied as a coating on pellets or tablets. COLAL-PRED^®^, a prednisolone metasulfobenzoate sodium-coated pellets formulation reached phase 3 clinical trials but failed to meet the primary endpoint, despite a superior safety profile. Further development led to the concept of blending an enteric polymer (i.e., Eudragit^®^ S), dissolving in the distal small intestine, with high amylose starch (resistant starch) in a single layer embodiment, the Phloral™ technology [75,76]. This combination allows drug release to be mediated by pH and bacterial enzymes functioning as a fail-safe system. Superior colonic targeting in comparison to standard Eudragit^®^ S coatings has been demonstrated by gamma-scintigraphy [76], and recently Phloral™ coated capsules were used for fecal microbiota transplantation for *Clostridium difficile* infections, in a clinical setting [78].

The recently launched OPTICORE™ technology was developed in order to overcome the inherent variability in transit time and accelerate drug release once a pH or enzymatic trigger is initiated. It comprises a combination of enteric polymer and polysaccharide as an outer layer (Phloral™) and an inner alkaline layer that promotes faster coating dissolution [63,79,80]. This technology was a key enabler for the successful phase 3 study of a 1600 mg mesalazine product in mild-to-moderate ulcerative colitis patients, which led to the launch of Asacol^®^ 1600 mg (also marketed as Asacolon^®^ 1600 mg and Yaldigo^®^ 1600 mg), now marketed in multiple countries.

## 4. In Vitro Methodologies for pH-Dependent Colonic Delivery Products

### 4.1. Pharmacopeial-Based Methods

The United States Pharmacopeia (USP) does not state any general methodology to study the release from colon-targeting products. However, the dissolution method for mesalazine delayed-released tablets includes a pH gradient. The assay starts by exposing the tablets to hydrochloric acid 0.1 N for 2 h, followed by a transfer into pharmacopeial phosphate buffer 159.3 mM (total phosphate concentration), pH 6.0 for one additional hour in the USP apparatus type II (37 °C, 100 rpm). Afterwards, a third dissolution stage is created in situ by adding a sodium hydroxide solution (to reach pH 7.2), while decreasing the paddle speed to 50 rpm [81]. Even though this method acknowledges the pH gradient from stomach to distal ileum, it does not accurately resemble other relevant aspects of physiology (such as buffer species and concentrations) and, therefore, it may not be suitable to predict the clinical performance of the product during formulation and development stages. This is of particular relevance for the dissolution of ionizable APIs and enteric polymers. Therefore, more bio-relevant methods and buffer systems are needed.

**Figure 3:**
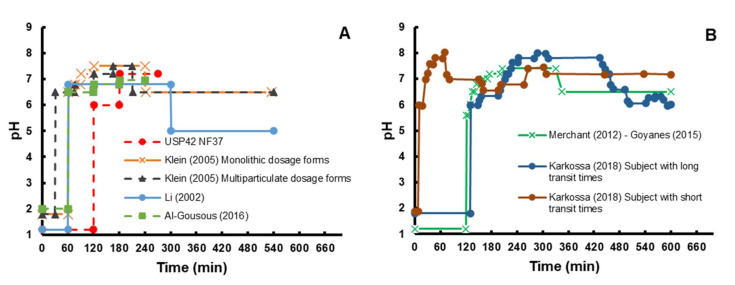
Examples in literature of in vitro pH gradients applied to resemble gastrointestinal gradients in bicarbonate-free (**A**) and bicarbonate-based set-ups (**B**). Further description of buffers is shown in Table 2 and in the respective references [81,83,84,85,86,87,88].

The USP type III apparatus appears to be advantageous to account for the GI environment gradient in a straightforward and robust fashion since pH gradients can be easily implemented, and its utility was shown by the IVIVC obtained by Klein et al. for a prototype colon-targeted formulation of caffeine [82]. Li et al. [83] compared the dissolution of paracetamol tablets coated with a three-polymer layer in the apparatus II and III using a buffer gradient that included the drop in pH after reaching the colon (Figure 3). The onset of dissolution was the same in apparatus II (900 mL, 100 rpm) and III (250 mL, 15 dpm, mesh 40) and coincided with the transfer into colonic media, which was citric acid/sodium phosphate dibasic pH 5.0 (Table 2) [83]. Hence, the impact of buffer composition on the in vitro dissolution was greater than the different hydrodynamics provided by the respective apparatuses. Interestingly, the reduction of the reciprocation rate or the mesh number, decreased the mechanical stress and, thus, the dissolution rate [83], suggesting that the interplay between dissolution media composition and apparatus parameters play a significant role in dissolution.

Marketed mesalazine and budesonide colonic-delivery products were studied in the reciprocal cylinder using a pH gradient based on compendial buffers (Figure 3, Klein et al.) [84]. The gradient applied highlighted product-related differences in drug release, which were consistent with the physical-chemical properties of their coating materials [84]. The same method was successfully used to study the in vitro release of caffeine (rapid and complete site-independent absorption) from granules coated with one layer Eudragit^®^ FS 30D (control) or two layers (Eudragit^®^ FS 30D and an additional inner layer of Eudragit^®^ RL:RS 2:8) [82]. The in vitro release from the latter product was not only slower, but also correlated well with the fraction absorbed obtained by deconvolution of plasma profiles from volunteers [82,89]. However, the in vitro dissolution occurred faster than the deconvoluted dissolution, which is most likely consequence of the extremely high buffer concentrations in compendial media compared to in vivo buffers, i.e., bicarbonate.

All compendial dissolution apparatuses (USP Type I, II and III) display design-related shortcomings to mimic colonic physiology. On the one hand, both the basket and the paddle apparatuses work with large volumes in order to provide sink conditions in the dissolution experiment. However, the volume used with USP type I/II is enormous compared to the volume of 49 mL determined in the ascending colon [33]. The reciprocated cylinder shows a slight advantage in this regard, but working volume is still at least four-fold higher than colonic fluid. This overestimation may be problematic not only for coating dissolution, but also for high dose/low permeable APIs such as mesalazine, where a physiological sink is not granted. On the other hand, mechanical stress applied in a pharmacopeial apparatus is continuous, while GI pressures and tablet movement/transit are discrete processes in physiological conditions [32]. Garbacz et al. [99] developed a dissolution stress test device, consisting of a two-compartment sphere (one for the dosage form and the other with an inner balloon) attached to a rotary axis that lies on the USP type I/II dissolution vessel. Stress parameters, namely pressure and velocity, are controlled by inflating/deflating the inner balloon and rotating/resting the horizontal axis (such that the sphere rotates between exposition to air/media), respectively. Dissolution of mesalazine locally acting colon-targeting products in this apparatus was found to be influenced by the stress conditions and was different from the results obtained from the pharmacopeial USP II apparatus [100].

### 4.2. Methods Based on Biorelevant Buffers

In the last decade more attention has been given to bicarbonate-based buffers as a more bio-relevant option in dissolution testing, particularly for ionizable APIs and enteric coated drug products.

#### 4.2.1. Bicarbonate Buffer

Bicarbonate buffer involves the following equilibrium:

CO_2(aq)_ + H_2_O_(l)_ ⇌ H_2_CO_3(aq)_ ⇌ H^+^_(aq)_ + HCO_3_^−^_(aq)_ ⇌ 2H^+^_(aq)_ + CO_3_^2−^_(aq)_ (1)

The second ionization step is irrelevant throughout the physiological pH range and can be ignored as long as no alkaline earth metal ions are involved. While the pKa of the H_2_CO_3_ ionization itself is around 3.3, potentiometric titration gives a value of 6.1 due to the equilibrium with CO_2_, which is virtually unperturbed by the relatively slow titration process.

The peculiarity of the bicarbonate-CO_2_ buffer system is related to the CO_2_-H_2_CO_3_ interconversion. While proton transfer reactions occur over timeframes of micro- and nanoseconds, the hydration reaction has a mean time of several seconds and that of the dehydration lies within the centi-second order of magnitude [101].

Taking into account that typical diffusional times during dissolution are in the order of deci-seconds, it can be inferred that while proton transfer reactions are practically at equilibrium during dissolution processes, CO_2_-H_2_CO_3_ interconversion is not [101]. This sets bicarbonate apart from the other buffers, the action of which typically involves solely proton transfer reactions. This is because the effective pKa governing the buffering action at the interface between the dissolution medium and the dosage form/API will be a function of the diffusion rates of CO_2_ and H_2_CO_3_, which will in turn depend on the hydrodynamics and viscosity [96,101,102].

At any rate, this effective pKa will be lower than the potentiometric one and thus far from the pH of the targeted intestinal segment. The corollary of this is poor buffering by intestinal bicarbonate for a dissolving carboxylic polymer film (like an enteric coat) and, therefore, a large gap between this film’s interfacial pH and the bulk pH exists. This leads to a sluggish polymer dissolution despite the bulk pH exceeding the polymer’s nominal dissolution pH threshold, as it is limited by the poor interfacial buffering capacity of bicarbonate (at the molarities present in intestinal fluid) [102]. Since the high buffer capacity compendial media poorly reflect this, they often tend to greatly overestimate the release rates from pH-dependent drug-delivery systems contributing to occasional in vivo product failure, particularly in cases where pH is not high enough or transit time is too fast [102]. Accordingly, at least during formulation development of pH-dependent drug delivery systems, it is important to use either physiological bicarbonate buffer or properly designed surrogates [96,102].

#### 4.2.2. Manual Methods to Stabilize Bicarbonate pH

Several studies support the in vivo predictive capacity of bicarbonate-based methods to assess the performance of pH-triggered release colonic products. Ibekwe et al. [103] compared the dissolution of three prednisolone tablets coated with Eudragit^®^ S in organic solution, Eudragit^®^ S in aqueous dispersion or Eudragit^®^ FS 30D in aqueous dispersion using different dissolution media. The Hanks buffer (bicarbonate-based) was able to discriminate between formulations at ileal pH 7.4, whereas similar dissolution profiles between the formulations were observed in phosphate-based media at the same pH. The onset of dissolution for these products was: Eudragit^®^ S_(aq disp.)_ < Eudragit^®^ FS 30D_(aq disp.)_ < Eudragit^®^ S_(org. sln)_ [103], and is thus consistent with observations in healthy humans using gamma-scintigraphy [59]. Moreover, the Hanks buffer accurately predicted the disintegration times, which ranged 195–305 min and 130–390 min, in vitro and in vivo, respectively [59,103]. This accurate matching is explained by the more bio-relevant bicarbonate concentrations in Hanks buffer, as well as the closer resemblance of luminal ionic strength and buffer capacity (Table 2) [90]. 

In spite of these advantages, the implementation of bicarbonate based-media into in vitro dissolution methods requires the handling of practical issues, for instance the pH instability during the experiment. The dehydration of carbonic acid rapidly generates carbon dioxide, which leaves the dissolution media as a gas, elevating the bulk pH. This instability may be even more critical when studying colon-targeted products, as longer dissolution experiments are expected. The equilibrium shifting towards carbon dioxide formation is further favored by the experimental conditions inherent to the dissolution assay, such as the temperature and media agitation. Continuous gas sparging has been used to overcome this issue and to maintain the bulk pH of bicarbonate buffer media. A mixture of CO_2_/O_2_ 5:95 enabled the pH stabilization of Krebs buffer (Table 2), which was used to study mesalazine release from commercial tablets coated with Eudragit^®^ S [90]. This method, however, was not able to stabilize the pH of Hanks buffer due to both the lower bicarbonate concentrations [90], and the fixed carbon dioxide partial pressure (P_CO2_) applied. Nevertheless, this latter condition can be controlled in the experimental set-up by tuning the ratio of CO_2_ to the inert gas, according to Henry’s law [104]. In this manner, gas flow rates can be manually adjusted before the experiment to achieve the desired pH. This technique was successfully used to isolate the contributions of the bicarbonate concentration and the bulk pH to drug release from enteric coated tablets [102]. By comparing the onset of release (t_20%_), it was concluded that bicarbonate conjugate base molarity had even greater impact on the pH-triggered dissolution of enteric coated tablets than the bulk pH. Therefore, the concentration of the buffer is a critical parameter when studying the release of colon-delivery products, based on pH-sensitive polymers, using in vitro methods.

The placement of physical barriers between the gas/liquid interface is an alternative approach to maintain the carbon dioxide concentration overtime. Fadda et al. [97] compared the dissolution of Asacol^®^ tablets in Krebs buffer at pH 7.4 stabilized by CO_2(g)_/air sparging, liquid paraffin layer on the media or sealing the vessel with an in-house developed lid made of nylon material. All these three pH-stabilizing methods were able to stabilize the pH in absence of drug products for 24 h and produced very similar dissolution profiles, which were much slower than dissolution rates in phosphate buffer [97]. Furthermore, the onset of dissolution matched the in vivo disintegration in humans determined by gamma-scintigraphy [105]. To the best of our knowledge, no commercial lid is currently available in the market, hence the application of this method requires the in-house manufacturing of the sealing lid. In this regard, any CO_2(g)_ impermeable material may be used. In fact, airtight sealing of the vessel does not seem entirely necessary. Recently, an in-house floating lid made of foamed styrol was utilized to stabilize the pH (ΔpH < 0.6 after 22 h) of bicarbonate 10 mM (pH 7.5) [106]. Even though the floating lid showed lower capacity to maintain the pH compared to its sealing counterpart, this method allowed discrimination between dissolution of three mesalazine colonic targeting coated tablets. Nonetheless, the lack of correlation against in vivo observations prevents any premature conclusion on the predictivity of this approach. Another sparging-free approach was published by Scott et al., which resulted in a successful pH stabilization of modified Hanks (mHanks) buffer, while still supplementing the media with CO_2(g)_ [107]. The authors developed an enclosure lid that comprises a plate with two gas inlet holes attached to a ring-shaped bottom chamber with multiple orifices to allow an even gas distribution into the media. The lack of bubbling with this system allowed the reconciliation of bio-relevant bicarbonate buffer with bile salts, preventing foam formation (See Section 4.2.4) [107]. Carbon dioxide impermeable lids may be a good alternative over the continuous sparging method, as they are easier and faster to assemble [97,106]. On the other hand, the paraffin layer method has the advantage of being easily adapted to other apparatuses with different vessel geometries. Nonetheless, its application may be limited to hydrophilic compounds, since lipophilic drugs have the potential of being partitioned into the paraffin layer [90]. A potential disadvantage of such physical barriers may be encountered under non-sink conditions since CO_2_ movement between gaseous and aqueous phases is an important component of its bulk buffering capacity [27].

#### 4.2.3. Automated Methods to Stabilize Bicarbonate pH

The implementation of CO_2(g)_ sparging testing in the pharmaceutical industry and for regulatory applications may be possible, provided an improvement in the method robustness and automatization (for instance, avoiding the manual tuning of carbon dioxide pressures applied). To that end, automated systems were developed. One example is the pHysio-stat^®^, an automated and stand-alone system that monitors and regulates the pH of the bicarbonate media by handling the CO_2(g)_ sparging [108]. The device consists of a pH electrode, a digital microcontroller, a valve system and a gas diffuser. Both the electrode and the diffuser are immersed in the dissolution media throughout the assay. The rise in bulk pH (as a consequence of losing CO_2(g)_) is sensed by the electrode and triggers a feedback mechanism, resulting in gas sparging (e.g., a mixture of CO_2_/N_2_ 1:9 *v*/*v*) into the media to restore the set pH [108]. The system was very accurate at maintaining the pH (±0.05 pH unit) of Hanks, Krebs and bicarbonate 5.95 mM under different conditions of rotational speeds (rpm) and operating pressures. Moreover, it was able to rapidly stabilize the bulk pH within 60 s after the addition of small amounts of sodium hydroxide (75 µmol). The device was successfully coupled with the USP type II apparatus to assess the effect of buffer concentrations and ionic strength on the dissolution of commercial enteric coated acetylsalicylic acid (ASA) [95], omeprazole and rabeprazole products [109]. Furthermore, it was possible to adapt this system to a miniaturized dissolution apparatus in an attempt to simulate the dissolution of extended-release valproate minitablets for pediatrics using CarbSIF media [110].

**Figure 4:**
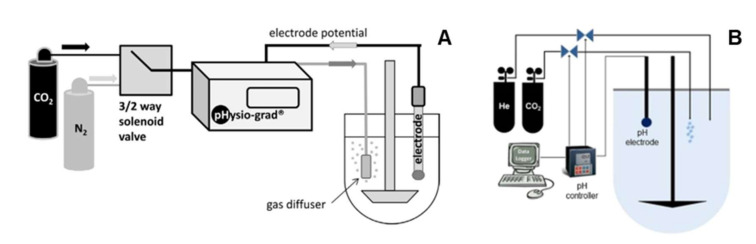
Schematic representation of automated bicarbonate systems pHysio-grad^®^ (**A**) and Auto pH^TM^ (**B**). Reproduced with permission from [111], Elsevier, 2014, and from [86], Elsevier, 2015, respectively.

Even though the aforementioned examples showcase possible applications of the pHysio-stat^®^, its static nature hardly resembles the dynamic conditions along the GI tract. Hence, a dynamic version was developed by coupling a second valve with the existing automated system, the pHysio-grad^®^ (Figure 4A) [111]. This allows CO_2(g)_ and an inert gas (e.g., N_2_ or He) to be separately sparged, such that the pH gradient can be set by modifying the flow rate ratio. Based on a pH gradient scheme previously designed by Klein et al. (Figure 3) [84], the pHysio-grad^®^ was used to study the dissolution of Nexium^®^ (enteric-coated pellets of esomeprazole) in Hanks buffer. The onset of dissolution was delayed when the gradient was applied, although the dissolution rate was steeper [111], most likely due to the constant gas sparging to maintain the bulk pH. The automated system produced a slower onset of dissolution in Hanks buffer compared to the USP phosphate buffer, applying the same pH gradient manually (by adding sodium hydroxide) [111]. Likewise, the in vitro dissolution of monolithic mesalazine locally acting colonic delivery products was delayed in CarbSIF (pH gradient handled by the automated system) compared to phosphate with the same gradient, revealing differences in the in vivo performance between Salofalk^®^ and Claversal^®^ coated tablets [88]. The ability to replicate pH gradients during dissolution can be very valuable to assess the robustness of drug products in different scenarios where intestinal pH and transit may change significantly. This variability can be due to age, gender and gastrointestinal diseases, such as inflammatory bowel disease, for instance [112,113,114].

A similar multiple-valves automated (Auto pH^TM^) system was developed and showed a tolerance of ±0.01 pH units (Figure 4B) [85,115]. Unlike the pHysio-grad^®^, the Auto pH^TM^ system was validated with a different methodology that includes an in situ change in buffer concentrations. Typically, after a gastric stage (2 h), the test product is transferred into 950 mL of modified Hanks buffer (mHanks [91], Table 2) for 35 min using the USP apparatus type II at 50 rpm. This modification relied on continuous CO_2(g)_ sparging to reach pH 6.8, which not only reduced the pH but also slightly enhanced the bulk buffer capacity from 1 to 3.1 mM/L/ΔpH [91]. After the proximal small intestinal stage, 50 mL of a “pre-Krebs” solution was added to the vessel in order to form a modified Krebs buffer (mKrebs) in situ (Table 2). Meanwhile, the bulk pH gradient was handled by the automated system to resemble both distal small intestinal and the colonic environments (Figure 3, Merchant–Goyanes) [85,115]. The method was able to recreate luminal buffer capacities of 3.1, 5.5 and 13 mM/L/ΔpH, for the upper small intestine, lower small intestine and colon, respectively (See Section 2) [91,97,115]. Furthermore, in situ transition from mHanks to mKrebs is key to better simulating the increasing bicarbonate concentrations upon the ileum. Using this method, the in vitro onset of dissolution for prednisolone tablets double-coated with Eudragit^®^ S (Duocoat^TM^) closely matched the in vivo disintegration times [115]. Likewise, mesalazine dissolution from different colon-targeting products correlated with in vivo disintegration times, in accordance with respective coating materials [86]. These examples, as well as several reports in the last years endorse the usefulness of this method predicting the in vivo release of novel pH-dependent colonic targeting formulations [116,117,118,119].

The aforementioned studies have strongly relied on the automated systems to simulate the dynamic environment along the GI tract in assessing the dissolution of pH-triggered colonic targeted products. However, limitations associated with this system are its expensive implementation for academic and regulatory purposes, as well as the extra efforts in buffer preparation and handling. These also limit method transferability and simplicity, which are key for quality control purposes. Alternatively, the dynamic CO_2_ and air gradient may be also adjusted manually throughout the experiment, in a similar fashion as mentioned in the previous section. However, the lack of a pH feedback mechanism in manual settings demands a proper pH vs. time validation prior to any dissolution experiment, together with a thorough pH monitoring throughout experiments.

#### 4.2.4. Bicarbonate-Free Bio-Relevant Methods

The CO_2(g)_ sparging itself may become another limitation when using bio-relevant bicarbonate-based media. The examples mentioned in the latter section often required large fluid volume, since experiments were typically performed in 900–1000 mL of bicarbonate-based buffer [108,111,115]. However, the gas sparging could positively impact on the media hydrodynamics, with the magnitude of this effect being greater in smaller volumes. In addition, bubbles may coat dissolving drug products, reducing the effective surface area available for dissolution. Furthermore, the design of some devices does not allow for inserting a pH-probe like the case of the USP type III apparatus. Even though the reciprocating cylinder apparatus has been underutilized, this is an attractive candidate to study colonic delivery due to the feasibility for creating bio-relevant gradients for pH and concentrations of luminal contents (e.g., bile, bacteria, enzymes, fatty acids, etc.) because of its ability to simulate the conditions within multiple GI segments in one experimental run. In addition, CO_2_ sparging is problematic when surfactants are needed in the media due to foam generation [120].

One set of bicarbonate-free media is the FaSSIF-related series, including fasted state simulated media resembling gastric fluid (FaSSGF), various versions of fasted state simulated intestinal fluid (FaSSIF-V1, -V2 and V3), ileal simulated intestinal fluid (SIF_ileum_) and ascending colon fluid (FaSSCoF) [92,94,98,121]. Those media are characterized by the presence of bile surfactants and buffer capacities lower than those of the compendial buffers, although they still tend to be too high compared to intestinal fluids (Table 2) [96]. The requirement for bile salt inclusion in the buffer may depend on the active ingredient properties. For instance, the pH gradient developed by Klein et al. [84] (Figure 3) was used to study the effect of bile salt-containing media (e.g., FaSSIF) on the dissolution of the mesalazine and budesonide marketed colon targeting oral products using the USP type III. This apparatus allowed for the decrease of luminal bile acids in more distal intestinal segments to be accounted for [34,35]. The authors found no significant effect of bile salts contained in these media upon drug dissolution [84], which is in good agreement with previous observations in a single-stage experiment using the apparatus II (FaSSIF vs blank-FaSSIF) [122]. Moreover, dissolution of mesalazine tablets coated with Eudragit^®^ FS 30D and Eudragit^®^ S was unaffected by taurocholate and/or lecithin [122]. However, this may be a consequence of the still high concentration of phosphate conjugate base in FaSSIF, [92] together with the hydrophilic nature of mesalazine [123].

However, some bio-relevant aspects that affects the solubility should be considered for poorly soluble drugs [94]. In fact, the solubility of ketoconazole, danazole and felopidine in human aspirates from the ascending colon was better predicted in FaSSCoF (bile acids, 0.15 mM; phosphatidylcholine, 0.3 mM, and bovine serum albumin, 3 mg/mL) compared to the blank media [98]. A level A IVIVC for MR capsules containing the poorly soluble weak acid, diclofenac, was successfully achieved by applying a gradient (Figure 3, Klein) that mimics the decrease in luminal bile salt concentrations into the apparatuses III and IV [124]. Although this example further supports the consideration of bile salts in in vitro set-ups, the predicted dissolution rate was still faster than the in vivo absorption, most probably due to the higher buffer molarities in both FaSSIF and SCoF (Table 2).

An elegant alternative to circumvent this issue may be through matching the dissolution medium performance to that of a relevant bicarbonate buffer based on the bicarbonate effective pKa (pKa_eff_) concept (i.e., surrogate buffer development). Even though accurate calculations of bicarbonate pKa_eff_ can be achieved for small molecules [125], the physical-chemistry of ionizable polymers (e.g., relaxation, swelling, disentanglement, multiple ionizable sites, among others) hinders any mathematical approach to calculate the pKa_eff_ and subsequently to design appropriate surrogate media. Nonetheless, semi-empirical approaches have been successfully developed. For instance, low molarity phosphate buffer (15 mM, pH 6.5) with an in situ gradient (Figure 3, Al-Gousous) was able to match the disintegration of ASA tablets enteric coated with either polyvinyl acetate phthalate (PVAP) or methacrylic acid-ethyl acrylate 1:1 copolymer in bicarbonate 8 mM media, as reference media. This method accurately predicted the pharmacokinetic parameters of these products obtained by deconvolution of healthy subjects’ plasma profiles [87]. Although useful, this approach strongly depends on the test product because the bicarbonate pKa_eff_ will vary according to the viscosity of the gel layer formed after the disentanglement of the coating material [96]. Recently, a more mechanistic approach for selecting a surrogate media was proposed and the in vitro dissolution of several products with diverse types of coating in different buffer species under bio-relevant conditions (molarities, temperature and ionic strength) was presented. The times for onset of dissolution in bicarbonate buffers tended to be between the times observed in experiments using succinate (pKa 5.2) and citrate (pKa 5.7) buffers. Based on these findings, the authors recommended a decision tree for selecting the most appropriate surrogate buffer. This involves an early assessment of the dissolution in bicarbonate and succinate, followed by only a few fine-tuning experiments depending on differences between dissolution in the surrogate media and in bicarbonate buffer (used as reference) [96].

## 5. In Vitro Methodologies for Time-Controlled Release Products

No general pharmacopoeial method exists for this type of formulation. For extended release mesalazine capsules, the USP specifies two tests: one involving only 50 mM pH 7.5 buffer for 8 h, and the other involving a 2 h stage in 0.1 M HCl followed by a 7 h stage in a 50 mM pH 6.8 phosphate buffer. Both tests can hardly be considered bio-relevant. 

As mentioned in Section 3, drug delivery of time-controlled products is based on the slowing down drug release so that only a minor portion is released before reaching the colon. This relies on the fact that colonic residence time typically constitutes the majority of the overall GI transit time and assumes a limited variability in gastric and small intestine transit times. Therefore, consideration of transit times throughout different segments of the GI tract is critical in developing relevant in vitro methodologies to assess the delivery from these types of vehicles. For instance, gastric residence time of Asacol^®^ (monolithic dosage form) was highly variable between 0.2–2 h, probably dependent on phase III waves of the MMC. Conversely, the release from Pentasa^®^, ethylcellulose coated microgranules, was faster between 0.2–0.5 h [126]. This variability was taken into account by Klein et al. [84], in an in vitro study on the release of locally acting colonic delivery systems using the USP type III apparatus, where the gastric phase was set to 60 or 30 min for monolithic or multiparticulate systems (Figure 3).

A more comprehensive analysis of transit variability effects was performed by Karkossa and Klein, who studied the in vitro dissolution of different mesalazine products in a multistage system using CarbSIF media [88]. They optimized transit time vs. pH gradient to account for different physiological scenarios previously observed (Figure 3, Karkossa et al.) [4]. They found that in vitro conditions mimicking average physiology, longer stomach residence and longer small intestinal residence predicted more than 60% of the dose from multiparticulate systems being released before reaching the ileocecal passage. This suggests a risk of dose dumping for these dosage forms, which may not exist in subjects with shorter gastric and small intestinal transits [88]. Accounting for physiological variability may be valuable in early screening for this potential risk in novel formulations, especially for those relying on GI transit and pH to elicit drug release.

Another issue is related to drug concentration within the core. This concentration determines the diffusional gradient driving the mass transport into the release medium and is a function of the drug solubility in the medium. Consequently, when dealing with an ionizable drug, it is important for the chosen buffer to achieve drug solubility similar to the in vivo situation. One example of the significance of this issue is the behavior of Pentasa^®^ in different media. In this case, bicarbonate-based intestinal stages led to lower dissolution rates than transfer into either USP phosphate or FaSSIF [84,86,88].

A different scenario applies for time-controlled release products containing neutral APIs, such as Entocort^®^ (Table 1). Budesonide early release in the stomach is prevented by the Eudragit^®^ L coating. In phosphate media, similar dissolution profiles were observed at pH 6.8 and 7.5. Furthermore, 70–80% of budesonide dose was consistently released within 3 h post-gastric stage (small intestinal transit) in diverse multi-stage in vitro methods that involved compendial, bile salt-containing [84], and bicarbonate-based media [115]. Of note, bile salts did not affect the release of budesonide from ethylcellulose-coated systems [84]. Even though bile salts may be able to cross the ethylcellulose coating, no effect on solubility is expected, as only monomeric bile salt fraction would reach the API surface. Therefore, the effects of bile salts on these systems may be limited to either ion pair formation with cationic drugs (decrease of solubility) or increasing the wettability for high contact-angle solid materials.

Some systems with time-dependent delivery mechanisms also include a pH-dependent delayed release component. For instance, Eudragit^®^ L coating is used to prevent gastric and proximal small intestinal release for some mesalazine products (Table 1). In this case, concerns expressed earlier regarding pH-dependent systems also apply. For instance, Mezavant^®^ showed no stomach release, followed by an apparent zero order kinetic (i.e., constant release rate) in phosphate buffer 50 mM in a gradient method attributed to the viscous layer formed after the dissolution of the MMX^TM^ (Multi Matrix System) coating [97]. By contrast, bicarbonate media revealed a 3 h lag time before starting diffusion followed by a relatively quick sigmoidal release curve [97], which actually corresponds to the available in vivo data [105,127]. Similar in vitro behavior was observed for Claversal^®^ micropellets and Salofalk GranuStix^®^ [88].

## 6. In Vitro Methodologies for Enzymatic-Triggered Drug-Release Systems

Besides all the relevant GI physiology aspects to consider in the setup of an appropriate dissolution system, as previously described, the enzymatic activity in the relevant sections must be considered to support the development of microbiota (enzymatic triggered) drug release systems. It is important to highlight that for the purpose of colonic targeting, polysaccharides are mostly used; therefore, the focus of this section lies on use of in vitro systems employing a medium containing bacterial enzymes able to degrade polysaccharides, such as starch.

To support the formulation development activities a dissolution method should have sufficient discriminatory power, but should also have sufficient robustness in order to allow meaningful comparison between batches and to better understand the impact of formulation/process variables on enzymatic drug release. There is a significant inter- and intra-individual variability in GI physiology [3], which ultimately makes the development of bio-relevant dissolution systems very challenging. Even more challenging are the efforts to reproduce in vitro the complex enzymatic composition found in GI fluids. Multiple dissolution approaches to characterize enzymatic-triggered drug release have been described. The simplest approach consists in the addition of selected commercially available enzymes to simulated buffers resembling the small intestine and the colon. Porcine pancreatin is commonly used to simulate the pancreatic enzymes flowing into the duodenum to demonstrate robustness of the dosage form to enzymatic degradation during transit in the small intestine [74,128,129]. Dissolution systems employing buffers supplemented with commercial bacterial enzymes could offer a relatively straightforward approach to evaluate enzymatic triggered drug release, in vitro. Enzymes, such as α-amylase (from *Bacillus licheniformis*, *Bacillus* sp., *Bacillus amyloliquefaciens*, *Aspergylus oryzae*) have been used for testing dosage forms containing resistant starches. Amylase from *Bacillus licheniformis* was shown to be the most promising enzyme in digestion of amylose in in vitro dissolution experiments [51].

The microbiota in the human colon largely outnumbers the total human cells [39], therefore the catabolic potential cannot be accurately represented by a restricted selection of enzymes and specific concentrations. Furthermore, over 50% of colonic bacteria species produce starch-degrading enzymes [130,131]. In order to simulate the complex colonic environment in terms of bacterial population, an in vitro medium containing selected bacterial strains (e.g., *Bifidobacterium* [129] or some members of the *Firmicutes* and *Bacteroidetes* phyla, with five potential candidates identified by Wahlgren et al.) [132] or complete fecal material has been proposed [51,128,129,133,134]. These can be produced out of fecal material from animal models, from healthy human subjects or from patients, in case a particular disease can impact the intestinal flora [112,135].

The composition of the fecal slurry is based on a method described by Hughes et al. [133] and further optimized by Basit and colleagues [134,136]. In brief, a basal medium [133] to allow bacterial growth is prepared and mixed in 1:1 ratio with a fecal slurry, which was prepared by homogenizing fresh human feces (pooled from three different healthy donors) in phosphate-buffered saline (pH 6.8) at a concentration of 40% *w*/*w*. The final concentration of the prepared fecal slurry (diluted with basal medium) was 20% *w*/*w*. Dosage forms were then tested in a predefined volume of fecal slurry, adjusted to the required pH, under continuous stirring. The tests were carried out in an anaerobic chamber (at 37 °C and 70% RH to ensure the growth and survival of anaerobic bacteria). Samples were then collected overtime for subsequent drug-release quantification. Due to the complex matrix effect, more effort is needed to develop an analytical method allowing a good peak separation. It is also key to understand whether the API is stable under these conditions in order to enable a good understanding of the release kinetic of the API out of the dosage form and avoid misinterpretations due to fast degradation of the API. 

**Figure 5:**
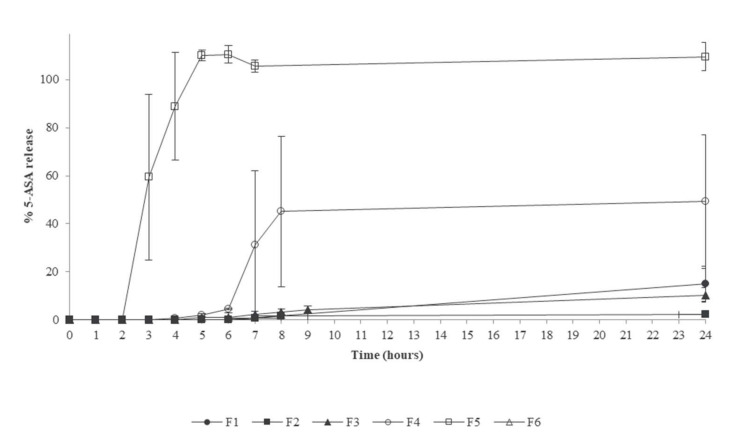
Drug release in human fecal slurry from Phloral™ coated tablets containing resistant starch processed at increasing temperatures: 25 °C (F1), 50 °C (F2), 60 °C (F3), 75 °C (F4) and boiling (F5). For comparison drug release from Eudragit^®^ S coated tablets is shown (F6). Adapted with permission from [75], Elsevier, 2020.

While the preparation of the fecal slurry medium is laborious and demands specialized equipment, it offers the possibility to assess whether enzymatic-triggered drug-release systems can effectively allow drug release due to bacterial enzymatic action. A positive impact of temperature during the processing of resistant starch used in the Phloral™ coating technology was observed using a human fecal slurry system (Figure 5) [75]. However, one needs to consider that bacterial populations along the large intestine are distinct and the fecal bacteria resembles rather the distal colon and rectum. Polysaccharides which escape digestion in the small intestine are mostly digested/fermented in the ascending colon, while protein digestion (i.e., from mucins) are digested more distally. Therefore, the bacterial population distribution depends on the substrates available. In order to facilitate and to standardize the preparation of the fecal slurry, it has been shown that fecal material can be frozen and stored at −70 °C and, thereafter, be thawed to be used without impairing enzymatic activity [137]. This process then allows to use the same batch of fecal slurry for multiple testing, reducing inter-test variability.

**Figure 6:**
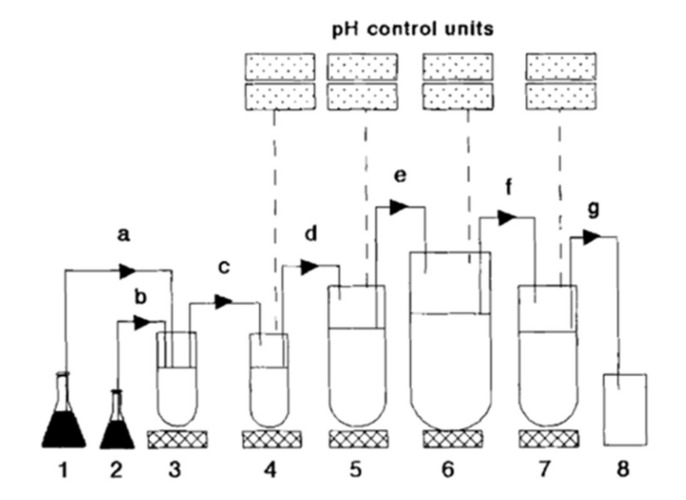
Diagram representing the SHIME (Simulator of the Human Intestinal Microbial Ecosystem) reactor. 1, feed; 2, pancreas acetone powder; 3, reactor 1 (duodenum and jejunum); 4, reactor 2 (ileum); 5, reactor 3 (caecum and ascending colon); 6, reactor 4 (transverse colon); 7, reactor (descending colon); 8 effluent pumps (**a**–**d**) worked semicontinuously; pumps (**e**–**g**) worked continuously. Reprinted from [138], Taylor & Francis, 1994.

Another tool available which employs human fecal material to recreate the microbiota environment in the human colon is the SHIME (Simulator of the Human Intestinal Microbial Ecosystem) system, offered by ProDigest (Figure 6). This system simulates the complete GI tract, including the different regions of the colon, where luminal conditions can be set to mimic the different pH and bacterial populations [138,139]. The colonic compartment is inoculated with human fecal material and the bacterial colonies are allowed to fully mature by a continuous nutrient feeding system. This allows long term intervention studies [140,141], but also can be used to investigate the impact of microbiota on dosage forms dissolution and API degradation. Additionally, short-term studies can be run with samples from the long-term incubators.

**Figure 7:**
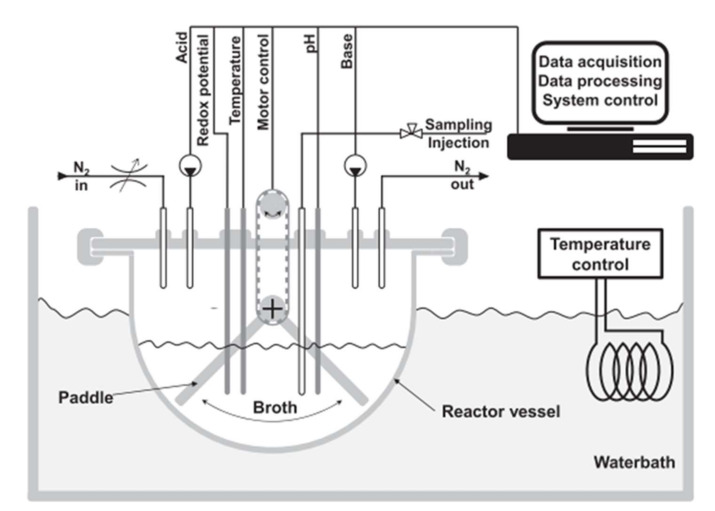
Diagram representing the Mimicol ascending colon system. Reprinted with permission from [142], Elsevier, 2021.

Despite the advantages of this system, it does not consider the dynamic environment in terms of nutrient availability from the digestion process and ascending colon simulation, where drug release should ideally be initiated. Very recently, a new system (Mimicol) was introduced which attempts to address the dynamic nature of the human colon by simulating more dynamic and physiologically relevant conditions (Figure 7) [142]. In this system, the medium (Chaedler broth) is inoculated with a sample complex microbiota pool derived from a fecal sample cultivation for 24 h. The medium is changed over time to allow continuous bacterial growth, and redox potential and pH are continuously monitored. The system also includes a mixing element design to simulate the peristalsis in the colon and the volume is kept at 150 mL. The dynamic ascending colon conditions simulated with the Mimicol system resulted in twofold increase in sulfasalazine degradation in comparison with a static batch fermenter [142].

**Figure 8:**
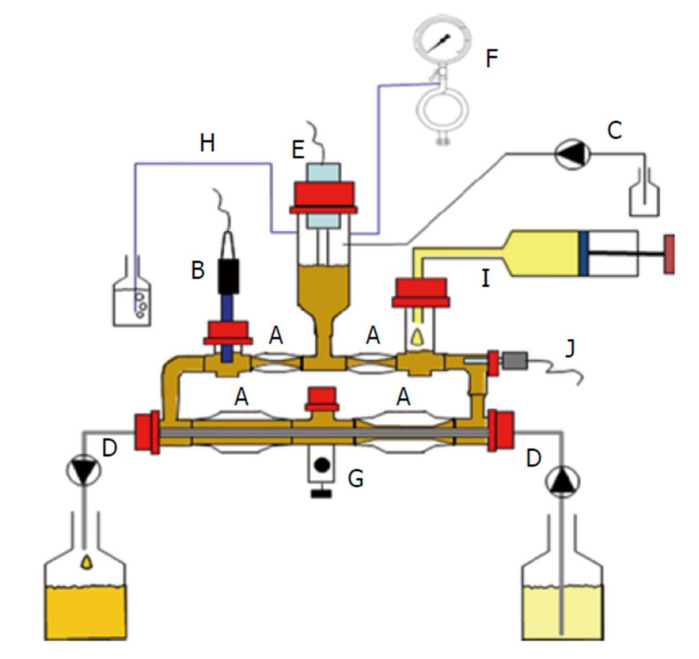
Schematic representation of the TIM-2 system. (**A**): Peristaltic glass unit.; (**B**): pH-monitoring unit; (**C**): pH-controller system (alkali pump); (**D**): Semi-permeable dialysis membrane; (**E**): Liquid volume sensor; (**F**): N_2(g)_ inlet; (**G**): Sampling port; (**H**): Gas outlet; (**I**): Inlet system for content emptied from ileum; (**J**): Temperature sensor. Reprinted from [146], Baishideng Publishing Group, 2014.

An even more complex system is the TIM-2 developed by TNO and now commercialized by the TIM company (Figure 8). This system aims at mimicking the colonic microbiota environment and can be used to assess the impact of colonic bacteria on food or drug products and the impact on microbiota composition after interventions with, for instance, prebiotics/probiotics [143,144]. The bacteria medium is fed with nutrients and absorption of metabolites is simulated by dialysis membranes. The system is fully computer-controlled in terms of peristaltic mixing, pH, temperature and volume [145]. The system can be used stand-alone or in combination with the TIM-1 model which simulates the gastric and small intestinal compartments. Its cost, complexity and the time required for microbiota culture stabilization limits its use as a screening tool during development and is mostly used for selected types of studies where a close simulation of the human colon is required. Additionally, similar to the Mimicol system, the throughput is low, and therefore cannot be used for screening purposes. In the case of these more specialized systems employing fecal material as a source of colonic bacteria, there is the difficulty in setting appropriate negative controls. In the case of colonic drug-delivery systems designed with an enzymatic trigger, it would be desirable to split between the enzymatic effect and other confounding factors which may interfere in the mechanism of coating dissolution, particularly if the film-forming polymer is pH-sensitive. Therefore, one strategy that can be followed is to use comparator formulations not containing the excipient used to induce drug release mediated by bacterial enzymes, given that in the absence of the bacteria or enzymes, no differences exist in drug release. However, this strategy demands an extra development effort. As described in this section, the simulation of the colonic bacterial environment is highly complex, difficult to standardize and, therefore, no compendial method is available. Hence, the dissolution methods for these types of colonic release systems need to be adapted to the particular needs.

## 7. Future Perspectives on In Vitro Testing for Locally-Acting Colon-Targeting Drug Products

When considering future perspectives for further development of dissolution testing methods for locally acting colon-targeted products, it is important to take into account that one-fits-all solutions are unachievable. Diligent and careful considerations should be made on the basis of the colon-targeting mechanism employed in the formulation, so the focus can be placed on the critical delivery parameters outlined in the previous sections. Accordingly, ideas for future perspectives regarding pH-triggered systems will differ from those conceived for enzyme-triggered systems.

Concerning in vitro methods, the different requirements of product development-related methods compared to QC methods should be taken into account. While a method guiding product development should provide as much bio-predictivity as possible, a QC method places much emphasis on simplicity, robustness and ease of transferability, which in practice requires a balanced sacrifice of bio-predictivity.

### 7.1. Methods for Formulation Development Purposes

In addition to the formulation principle-related considerations outlined in the previous sections, efforts should be made to increase patient centricity. This means methods should not be developed based only on the conditions in healthy humans, but also on those in the target population. Given that those locally acting products typically aim for treating GI disorders, where physiological conditions could be different, the importance of accounting for this aspect cannot be overstated. Ideally this will result in fewer surprises in clinical trials [112]. Beyond that, the specific technical considerations should be made for each of the main formulation approaches.

#### 7.1.1. pH-Dependent Systems

As displayed throughout this manuscript, the literature reviewed agrees that consideration of the unique buffering properties of bicarbonate is key to improving the predictions of dissolution for these dosage forms. Since the use of bicarbonate is associated with some technical difficulties, a practical approach, appropriate bicarbonate surrogate buffers (as previously described in this manuscript) could be employed for the majority of development iterations. However, for important milestone development steps, it might still be most appropriate to use bicarbonate-containing buffers.

#### 7.1.2. Time-Controlled Systems

For time-dependent systems, we suggest choosing buffers that result in a drug solubility inside the dosage form which can be considered as reasonably bio-relevant. Furthermore, any transit time variability needs to be accounted for in the experimental design setup. The pH gradient variations employed by Karkossa et al. [88] (Figure 3) may be a reasonable starting point to explore the effect of physiological variability on product performance.

#### 7.1.3. Enzymatic Triggered Systems

For those systems, isolated enzymes may be satisfactory for most development steps. Nevertheless, analogous to the situation with the pH-triggered systems, the use of more complex cultures and/or fecal slurries is strongly encouraged when it comes to major milestone steps during development.

### 7.2. Methods for Quality Control Purposes

The dearth of compendial methods for colon-targeted formulations is problematic. In the USP there are methods specific for formulated mesalazine preparation: The methods for the pH- and time-dependent systems suffer from the aforementioned shortcomings associated with compendial methods, while for enzyme-dependent products no methods are listed. This leaves much room for ideas regarding novel method development.

When it comes to QC methods, while bio-predictivity considerations are still important, they are not as crucial as in the case of methods employed during formulation development. Here, it should be of equal importance to create easy-to-use methods, while avoiding unnecessary steps that are prone to experimental error and showing high robustness and good reproducibility. The methods should be readily transferable, the material should be easily accessible, and the protocol and technology should be easy to learn for new employees to enable testing at different sites and in different environments, if needed. For a longer-term perspective, considering marketed products, the cost of goods and sustainability are subjects of discussion and should not be overlooked. These considerations shed light on the recurring dilemma of striking the right balance between method simplicity and degree of bio-predictivity. The discriminatory power should be reasonably similar to what is encountered in the in vivo condition, such that clinically relevant batch quality issues could be reliably detected while avoiding over-discrimination and unnecessary batch release delays. In the long run, it can be worthwhile to follow stepwise approaches gradually increasing experimental complexity until discrimination is deemed satisfactory, while the complexity is still not excessive.

#### 7.2.1. pH-Dependent Systems

It appears that bicarbonate-free surrogate buffers could be promising for introduction to quality-control testing. As shown throughout this review, even though pharmacopeial methods do account for physiological pH gradients, they still overlook critical parameters such as buffer capacity and molarity. This may result in a risk of producing a batch that passes QC, while still failing to achieve a satisfactory degree of colonic delivery [109]. In this regard, the most likely risk would be the manufacture of false positives, due to the abnormal concentrations used in pharmacopeial testing media. However, these surrogate buffers have low bulk buffering capacity that can be problematic when it comes to the high strengths of ionizable substances. In the absence of routinely used auto-titrators in dissolution testing, a compromise may need to be struck through enhancing the bulk buffering capacity to a minimal reasonable value. Furthermore, since coat robustness against mechanical stresses is important, adding an evaluation in a disintegration tester, where mechanical stresses are greater than in a dissolution tester, may be recommended.

#### 7.2.2. Time-Controlled Systems

For time-dependent systems containing ionizable compounds, striking the right balance between drug solubility in the core and ability to maintain the bulk pH is important. In addition, since mechanical robustness is important, adding an evaluation in a disintegration tester, where mechanical stresses are greater than in a dissolution tester, may be recommended (this applies regardless of compound ionizability).

#### 7.2.3. Enzymatic Triggered Systems

For these systems, the use of commercially available enzymes in dissolution media may be the best approach as it circumvents the complexity associated with bacterial cultures and fecal slurries. Enzyme selection will depend on the coating material. The same mechanical stability considerations as in the two previous classes apply.

## 8. Considerations for In Vivo Methods

It is generally recommended to employ region-specific authority guidelines for any bioequivalence testing of locally targeted dosage forms [147]. The FDA and EMA frameworks do differ in their principles, as the FDA framework is driven by product specific recommendations [148,149,150] whereas the EMA guidelines provide distinct recommendations for the demonstration of equivalence for locally acting products in the gastrointestinal tract in general [151,152]. Thus, the authorization pathways in both regions may be different depending on the product and are not harmonized [147].

The main problem with the current BE guidelines is their dependence on plasma concentration profiles as a gold standard. However, the use of plasma concentrations as surrogate markers for drug levels at the site of action is problematic in this case. The site of action for such locally acting products is reached before entering into systemic circulation. Therefore, especially when dealing with compounds having limited colonic permeability, plasma concentrations are far from optimal in reflecting what happens at the site of action. Different release rates and, therefore, different availability at the site of action could result in similar plasma concentration profiles i.e., we face an increased risk of bioequivalence overestimation. This is because the presence of additional barriers to mass transport between the local site of action and the systemic circulation can mask the differences observed between formulations when using plasma concentration as a performance marker. Therefore, plasma concentration data should be supplemented with in vitro bio-predictive dissolution experiments using bicarbonate solutions or fecal slurries (according to formulation type).

Moreover, one can use imaging methods, such as gamma-scintigraphy, magnetic marker monitoring and magnetic resonance imaging [19,79,153], as well as data on fecal concentrations as in vivo markers. However, many of these investigations are costly and time intense and, in the case of imaging technologies, do not provide information on the active moiety release but on the labelled moiety (but does provide information on the site of disintegration, however). As for fecal concentrations, they suffer from the problem of pooling over the many hours between defecation events. More meaningful data on the release kinetics can be gathered by intraluminal aspiration studies; however these are associated with caveats such as high costs due to procedure complexity, and general disturbance of the gastrointestinal environment due to the introduction of tubes. Therefore, while such additional in vivo procedures could be encouraged, making them mandatory may be excessive. From a business perspective, it may be appropriate to schedule these investigations prior to entering a pivotal study in order to preclude costly formulation bridging at a later stage.

## 9. Conclusions

Predictive in vitro methods for locally acting colonic-targeting products using pH-triggered, time-dependent and enzymatic-triggered release strategies were reviewed in this manuscript. The lack of bio-relevance of compendial methods limits their application during formulation development. The diversity in colon-targeting mechanisms demands a diversity of dissolution methods, with the compendia completely ignoring a whole class of formulations (enzyme-dependent). This may be due to relevant products not being on the market for a long time, the lack of experience in quality control settings, and limited experience in working with this type of products in the industry in general. Accordingly, greater efforts are required in academia and the pharmaceutical industry to develop sets of standardized methods that can be implemented with limited complexity and good robustness. Additionally, what makes the need for improvement particularly urgent is the fact that plasma concentration is often a poor bioavailability indicator due to its potential of being a poor indicator for this type of product. With the available in vivo evaluation approaches being expensive, laborious and suffering several shortcomings of their own, bio-predictive dissolution methods can occupy a special niche in terms of supplementing plasma concentration data for regulatory approval.

## Figures and Tables

**Table 1:** Locally-acting colonic targeting pharmaceutical products and their respective coating technologies.

Product	API	Dosage Form	Coating Polymer	Polymer Brand Name	Release Mechanism
Asacol^®^	Mesalazine	Delayed-release tablets	Methacrylic acid copolymer type B	Eudragit^®^ S	pH-dependent release (pH > 7.0)
Mezavant^®^/ Lialda^®^	Mesalazine	Delayed-release tablets	Methacrylic acid copolymer type A & B	Eudragit^®^ L Eudragit^®^ S	pH-dependent release (pH > 6.0) pH-dependent release (pH > 7.0)
Uceris^®^	Budesonide	Extended-release tablets	Methacrylic acid copolymer type A & B	Eudragit^®^ L Eudragit^®^ S	pH-dependent release (pH > 5.0) pH-dependent release (pH > 7.0)
Salofalk^®^	Mesalazine	Delayed-release granules	Methacrylic acid copolymer type A	Eudragit^®^ L	pH-dependent release (pH > 6.0)
Pentasa^®^	Mesalazine	Controlled-release capsules	Ethylcellulose Hydroxypropyl Methylcellulose	Surelease^®^	Time-controlled sustained release
Asacol^®^/ Yaldigo^®^/ Asacolon^®^ (OPTICORE^TM^)	Mesalazine	Delayed-release tablets	Methacrylic acid copolymer type B & Resistant Starch	Eudragit^®^ S	pH-dependent release (pH > 7.0) & Enzymatic triggered release

**Table 2:** Characteristics of in vitro compendial-based, bio-relevant and surrogate media for pH-triggered locally acting colonic products.

Buffer Name	Resembling Segment	Buffer Species and Molarities	Other Relevant Contents 3	pH	IS (mM)	Buffer Capacity (mmol/L/ΔpH)	Media Type ^a^
Hanks [90]	Proximal small intestine	Bicarbonate 4.2 mM ^b^	Mg^2+^, Ca^2+^	7.4	155	1	Bio-relevant
		Phosphate 0.3 mM ^b^					
mHanks [91]	Proximal small intestine	Bicarbonate 4.2 mM ^b^	Mg^2+^, Ca^2+^	6.8 ^c^	155	3.1	Bio-relevant
		Phosphate 0.8 mM					
FaSSIF (V-1) [92]	Proximal small intestine	Phosphate 28.7 mM ^d^	TC, LC	6.5		12 [93]	Bio-relevant
(V-2)		Maleate 19.1 mM ^e^				10 [94]	
CarbSIF [95]	Proximal small intestine	Bicarbonate 15 mM		6.0–6.8 ^c^	140		Bio-relevant
- [87]	Proximal small intestine	Phosphate 15 mM		6.0	139		Surrogate
- [96]	Proximal small intestine	Succinate 15 mM ^b^		6.8	139		Surrogate
- [96]	Proximal small intestine	Citrate 15 mM ^b^		6.8	139		Surrogate
Krebs [97]	Distal small Intestine	Bicarbonate 25 mM	Mg^2+^, Ca^2+^		161	3.7	Bio-relevant
mKrebs [97]	Distal small Intestine	Bicarbonate 25 mM	Mg^2+^, Ca^2+^	7.4 ^c^	161	5.5	Bio-relevant
SIF_ileum_ [94]	Distal small Intestine	Maleate 52.8 mM	TC, LC	7.5		10	Bio-relevant
- [87]	Distal small Intestine	Phosphate 19.5–23.5 mM		6.8–6.95	139		Surrogate
- [96]	Distal small Intestine	Bicarbonate 30 mM ^b^		7.4	139		Surrogate
- [96]	Distal small Intestine	Succinate 15 mM ^b^		7.4	139		Surrogate
-[96]	Distal small intestine	Citrate 15 mM ^b^		7.4	139		Surrogate
- [83]	Colon	Citrate		5.0			Compendial-based
		Phosphate					
SCoF [84]	Colon	Acetic acid 170 mM		5.8	160	29.1	Compendial-based
FaSSCoF [98]	Ascending colon	Tris 45.4 mM	BSA, PA, PC	7.8		16	Bio-relevant
		Maleate 75.8 mM					

^a^: Use of surrogate buffer depends on each product/coating and require previous validation against a bio-relevant bicarbonate-based media. Reader is referred to text and references for details. ^b^: Buffer concentrations based on the conjugated base molarity. ^c^: pH achieved by CO_2_/air sparging. ^d^: FaSSIF-V1. ^e^: FaSSIF-V2. IS: Ionic strength; TC: taurocholate; LC: lecithin; BSA: bovine serum albumin; PA: Palmitic acid; PC: Phosphatidylcholine.
